# Comparison of biofilm models for producing artificial active white spot lesions

**DOI:** 10.1590/1678-7757-2023-0458

**Published:** 2024-06-04

**Authors:** Erika Michele dos Santos ARAUJO, Cristina de Mattos Pimenta VIDAL, Min ZHU, Jeffrey A. BANAS, Anderson Zanardi de FREITAS, Niklaus Ursus WETTER, Adriana Bona MATOS

**Affiliations:** 1 The University of Iowa College of Dentistry Iowa Institute for Oral Health Research Iowa City IA USA The University of Iowa, College of Dentistry, Iowa Institute for Oral Health Research,Iowa City, IA, USA.; 2 Universidade de São Paulo Faculdade de Odontologia Departamento de Dentística São Paulo SP Brasil Universidade de São Paulo, Faculdade de Odontologia, Departamento de Dentística, São Paulo, SP, Brasil.; 3 The University of Iowa College of Dentistry Department of Operative Dentistry Iowa City IA USA The University of Iowa, College of Dentistry, Department of Operative Dentistry, Iowa City, IA, USA.; 4 The University of Iowa College of Dentistry Department of Pediatric Dentistry Iowa City IA USA The University of Iowa, College of Dentistry, Department of Pediatric Dentistry, Iowa City, IA, USA.; 5 Universidade de São Paulo Instituto de Pesquisas Energéticas e Nucleares Centro de Lasers e Aplicações São Paulo SP Brasil Universidade de São Paulo, Instituto de Pesquisas Energéticas e Nucleares (IPEN), Centro de Lasers e Aplicações, São Paulo, SP, Brasil.; 6 Universidade Católica Portuguesa Faculdade de Medicina Dentária Viseu Portugal Universidade Católica Portuguesa, Faculdade de Medicina Dentária, Viseu, Portugal.

**Keywords:** Dental caries, Dental enamel, Biofilms, Microbiology, Raman spectrum analysis

## Abstract

Creating artificial caries-like lesions that mimic the complex changes observed in natural caries is essential for properly evaluating new strategies, dental materials, and devices designed to arrest their progression and avoid more costly and invasive treatments.

**Objective:**

This study compared three protocols for developing artificial white spot lesions (WSL) using biofilm models.

**Methodology:**

In total, 45 human enamel specimens were sterilized and allocated into three groups based on the biofilm model: *Streptococcus sobrinus* and *Lactobacillus casei* (Ss+Lc), *Streptococcus sobrinus* (Ss), or *Streptococcus mutans* (Sm). Specimens were incubated in filter-sterilized human saliva to form the acquired pellicle and then subjected to the biofilm challenge consisting of three days of incubation with bacteria (for demineralization) and one day of remineralization, which was performed once for Ss+Lc (four days total), four times for Ss (16 days total), and three times for Sm (12 days total). After WSL creation, the lesion fluorescence, depth, and chemical composition were assessed using Quantitative Light-induced Fluorescence (QLF), Polarized Light Microscopy (PLM), and Raman Spectroscopy, respectively. Statistical analysis consisted of two-way ANOVA followed by Tukey’s *post hoc* test (α=0.05). WSL created using the Ss+Lc protocol presented statistically significant higher fluorescence loss (ΔF) and integrated fluorescence (ΔQ) in comparison to the other two protocols (p<0.001).

**Results:**

In addition, Ss+Lc resulted in significantly deeper WSL (137.5 µm), followed by Ss (84.1 µm) and Sm (54.9 µm) (p<0.001). While high mineral content was observed in sound enamel surrounding the WSL, lesions created with the Ss+Lc protocol showed the highest demineralization level and changes in the mineral content among the three protocols.

**Conclusion:**

The biofilm model using *S. sobrinus* and *L. casei* for four days was the most appropriate and simplified protocol for developing artificial active WSL with lower fluorescence, higher demineralization, and greater depth.

## Introduction

White spot lesions (WSL) are formed as bacteria from the cariogenic biofilm release acids, decreasing the pH of plaque and demineralizing the tooth.^1^Over time, the lack of mineral equilibrium between tooth, plaque, and oral fluids establishes a subsurface disorganization that results in a WSL. The dynamic processes of demineralization/remineralization and the characteristics of the enamel contribute to the final features of these lesions,^2^ which are notably subsurface demineralization and a more mineralized surface layer, while the partial dissolution of the hydroxyapatite crystals lead to enlargement of the intercrystalline spaces.^2,3^Clinically, the enamel presents a rough and white chalky surface, which affects esthetic outcomes in anterior teeth.

The progression of WSL can lead to a substantial loss of the tooth structure that requires more invasive and costly treatment, so lesions are best managed at their initial stage. Numerous reparative materials, remineralizing products, and diagnostic equipment and tools have been studied to detect lesions and arrest caries progression in its early phase.^4^ Several *in vitro* studies evaluating non-surgical approaches and arresting materials to manage WSL have been conducted by measuring the progression of artificial initial caries lesions obtained by chemical means that promote the dissolution of the enamel.^5,6^ However, the resulting lesions do not mimic the complexity of changes in tissue chemical composition and structural organization observed in natural caries lesions.^5^

In this context, *in vitro* models that employ cariogenic biofilms for long periods of time and under controlled laboratorial environment to create artificial lesions have been prioritized.^7-9^ These biofilm models are an excellent alternative for mimicking the natural condition of the oral cavity, studying biofilm formation, creating caries lesions, and developing and testing new therapies to arrest caries lesion progression.^10-12^ On the other hand, *in vitro* biofilm models also use a single strain, mainly *S. mutans,* within a pure culture system that leads to a mono-species biofilm.^13^ *In vivo* cariogenic biofilms are composed of numerous strains that cannot be readily replicated *in vitro*; however, multi-species microbial and microcosms approaches have been proposed for the development of artificial caries lesions.^7,9,10^ In this context, despite these more complex biofilm models producing artificial lesions similar to the natural ones, differences in protocols can impact the outcomes of studies evaluating lesion progression or arrest.

The main differences in the conditions used among the several biofilm models described in the literature are the bacterial species (single or multispecies), the continuous or intermittent exposure to sugar, the length of the protocol, and the culture medium flow (static or dynamic).^5,14^ Moreover, a potential disadvantage of these biofilm models is the creation of erosion lesions instead of a WSL due to surface softening and cavitation of the enamel.^15^ In this context, an important consideration when developing or optimizing *in vitro* protocols for developing artificial caries lesions is that some microbiological methods do not include remineralization.^15,16^ This step is essential for producing a WSL with the enamel surface intact to explore the inner features of the artificially created lesion.

Therefore, this study compared three biofilm models for developing WSL using single and combined bacterial species considered primary cariogenic microorganisms related to the development of caries lesions. The artificial WSL were investigated including lesion fluorescence, depth, and chemical composition. The null hypothesis was that all biofilm models would create active WSL with similar depth, as well as optical and chemical characteristics.

## Methodology

### Enamel specimen preparation

The study protocol was approved by the local Institutional Review Board (IRB #202304077). A total of 45 extracted human molars (n=15/group) were cut into 5×5 mm samples from the buccal enamel surface using a low-speed diamond saw under water cooling (Isomet 1000, Buehler, Lake Bluff, IL, USA). Then, the buccal surface was covered with acid-resistant nail varnish, exposing a 4×4 mm enamel area. All samples were sterilized using gamma radiation at 25kGy. In addition, natural WSLs were selected to be compared with the three studied protocols for artificial WSLs, as described below.

### Saliva collection and incubation

All participants, accounting for six healthy donors (three male and three female) (IRB #200805755), signed an informed consent form before saliva collection, which consisted of stimulated saliva using parafilm collected for 10 min. Then, saliva from all donors was pooled, diluted (1:1) with phosphate-buffered saline (PBS), and centrifuged at 3000×g for 10 min at 4°C. The resultant supernatant was filter-sterilized using a 0.22 μm general-purpose filter system (Corning, NY, USA), diluted again in PBS (1:4), and stored at −80°C. Before the protocol for creating the WSLs (described in detail as follows), all samples were immersed in 1.5 ml sterile saliva filtrate and kept under agitation for 1 h to form the acquired pellicle. After that, the saliva was removed and the samples were randomly allocated into the groups described in detail as follows.

### Biofilm models for creation of the artificial WSL

Streptococcus sobrinus (ATCC 33478) (Ss), Lactobacillus casei (ATCC 4646) (Lc), and Streptococcus mutans (ATCC 25175) (Sm) were the strains selected for the different biofilm models. Sm and Ss were tested individually, and Ss was also tested in combination with Lc. The individual strains were cultivated on blood agar plates (CDC formulation, Thermo Fisher Scientific, Remel) at 37°C and 5% CO_2_ for 48 h. After incubation, each strain was transferred to tubes containing of their specific medium supplemented with 1% sucrose, which were Lactobacillus MRS broth (MRS) (pH 6.2) for Ss+Lc, Tryptic Soy Broth (TSB) (pH 6.0) for Ss, and TSB (pH 6.3) for Sm. The average optical density (OD600) for all the groups was 0.4±0.05. Then, the media was used to incubate the enamel samples in the different groups. For all groups, cycles of demineralization (by incubating the enamel samples with the bacteria) followed by remineralization were performed every four days (three days of demineralization and one day of remineralization), with a total of four days (one cycle) for Ss+Lc, 16 days (four cycles) for Ss, and 12 days (three cycles) for Sm (n=15). After each three-day demineralization time, biofilm was removed from the enamel surfaces and samples were washed under agitation in Dulbecco’s phosphate-buffered saline (DPBS) for 1 h. After washing, samples were immersed in remineralization solution (1.5 mM CaCl_2_, 0.9 mM KH_2_PO_4_, 130 mM KCl, 20 mM sodium cacodylate, and 2 ppm sodium fluoride, pH 7)^17^ for 24 h at 37°C. The number of cycles for each protocol was selected based on a pilot study that determined the required incubation time to obtain WSLs without cavitation. Fresh media was renewed daily, and a new inoculum was prepared for each cycle for all models to ensure maximum bacterial growth. The final pH of each inoculum was monitored daily using a pH meter (Accumet AB150 pH meter, Thermo Fisher Scientific, Waltham, MA).

### Quantitative Light Fluorescence (QLF) analysis

After the creation of the WSL, the nail polish was removed from the surface, samples were dried for 10 s to distinguish between sound enamel and WSL, and QLF images were obtained using the QLF^TM^ System (Inspektor Research Systems BV, Amsterdam, NL). Then, QLF images were analyzed by a single examiner using QLF software (Inspektor^TM^PRO, Inspektor Dental Care, Amsterdam, NL). The fluorescence loss (ΔF) and integrated fluorescence (ΔQ) values were recorded, and the ΔF between sound enamel and WSL was quantified. The ΔF (%), area of the WSL (mm^2^), and the fluorescence integrated by lesion area (%mm^2^) were obtained for each sample and averaged in each group.

### Polarized Light Microscopy (PLM)

Samples were sectioned longitudinally using a hard tissue microtome (Hard Tissue Microtome, Series 1000 Deluxe; Silverstone-Taylor, SciFab, Lafayette, CO, USA) to obtain 15 sections (200–300µm) from each group, which were analyzed under a PLM (Olympus BX50, Olympus). Images of the cross-section were obtained using a camera attached to the microscope (Olympus DP72, Olympus), and the photomicrographs were taken at 10× and 20× magnification under illumination. For each image, lesion depth (µm) was measured by drawing five lines perpendicular to the outer enamel surface to the bottom of the lesion using the Image-Pro Insight Software (Media Cybernetics, Silver Spring, USA). The lesion depth was obtained for all samples and averaged in each group.

### Raman spectroscopy - biochemical analysis and mapping

The surface of representative WSLs and their adjacent sound enamel from three samples in each group were chemically analyzed using the LabRAM HR Evolution Raman spectrometer (HORIBA, Scientific France SAS, France). In total, 90 acquisitions of 1 s each were obtained at wavelength 785 nm, 120 mW, 0.7cm^-1^ of spectral resolution, a pinhole of 70 µm, 600 lines/mm reading grid, and a 10× objective. A total of three points were analyzed for each sample, and the reading range was 400–1500cm^-1^. Raman mapping was performed on the surface using a wavelength of 785 nm, 120 mW, three accumulations of 15 s each, 0.7cm^-1^ of spectral resolution, a pinhole of 70 µm, 600 lines/mm reading grid, and a 10× objective. Moreover, 36 (6 by 6) points were analyzed for each sample, where the central region of the surface containing both sound enamel and WSL was mapped. The reading range was 400–1500cm^-1^. After the reading, two spectra for each region (sound and WSL) were chosen as standard spectra, and two different colors were attributed for each substrate, namely blue (sound enamel) and red (WSL). All spectra underwent baseline correction using a 4-point third-order polynomial and smoothing using an 8-point second-order polynomial using LabSpec 6 (HORIBA, Scientific France SAS, France).

### Statistical analysis

Data were analyzed using Minitab 19 (Minitab, Pennsylvania, USA). Differences in lesion ΔF, ΔQ, and depth between the different biofilm protocols were compared using two-way ANOVA followed by Tukey *post-hoc* tests using a 95% level of significance (p<0.05).

## Results

**Figure 1:**
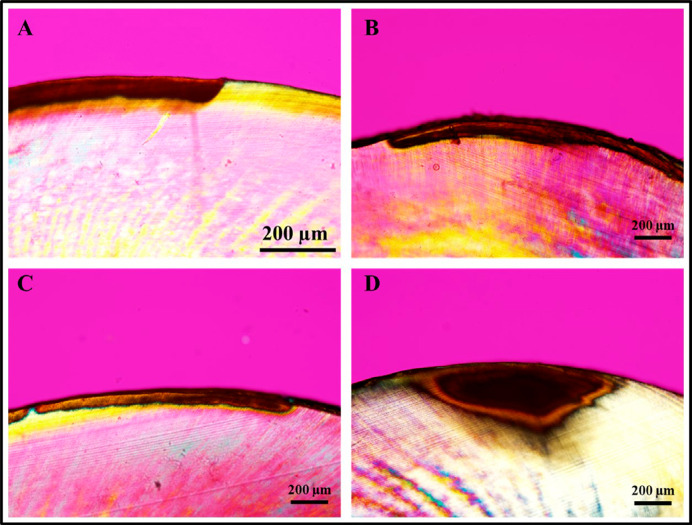
Polarized light microscopy images of the artificial white spot lesions (WSL) created on the enamel surfaces using the different biofilm protocols. Images were obtained under 20× (A) and 10× (B, C, and D) magnification. A: Ss+Lc, B: Ss, C: Sm, D: natural WSL (for comparison).

Table 1 shows the changes in pH of the demineralization solution (inoculum) during the demineralization cycles. The greatest decrease in the initial 24 h of incubation was observed for the Ss+Lc group, but both Ss+Lc and Ss groups presented low pH after 72 h (below 4), indicating great potential to induce demineralization. In addition, the Ss+Lc retained a steady low pH, whereas the Ss and Sm models continued to decrease. Table 2 shows the results for QLF measurements and lesion depth (obtained from PLM images). The Ss+Lc protocol produced lesions with a statistically significant change in the ΔF and ΔQ in comparison to the other two protocols (p<0.001). We found no statistically significant difference between Ss and Sm. Regarding lesion depth (Table 2 and Figure 1), the Ss+Lc protocol resulted in the deepest WSL followed by Ss and Sm. By comparing the artificial WSL to natural lesions in the extracted teeth, we found that none of the protocols created lesions with similar depth to the natural ones (Figure 1). For all PLM images, it is possible to observe the body of the lesion and the surface layer. When examined in water, all PLM images showed the demineralized area (body) positioned in the subsurface of enamel, characterized by a positive birefringence and covered superficially by a slightly negative birefringent surface layer, relatively unaffected by the bacteria as showed in Figure 1. However, none of the artificial WLS mimic the shape of the natural WSL (convergent to the dentin-enamel junction), as depicted in Figure 1D.

**Table 1:** Changes in pH during the three days of the demineralization cycles using the different biofilm models. Ss: *Streptococcus sobrinus*, Lc: *Lactobacillus casei*, and Sm: *Streptococcus mutans*.

Groups	pH			
	Initial	24 h	48 h	72 h
Ss + Lc	6.2	3.7	3.7	3.7
Ss	6.2	4.8	4.7	3.9
Sm	6.3	5	4.5	4.3

**Table 2:** Results (mean±standard deviation) of fluorescence loss (ΔF), integrated fluorescence loss (ΔQ), and lesion depth for all the biofilm-induced protocols (n=15). Ss: *Streptococcus sobrinus*, Lc: *Lactobacillus casei*, and Sm: *Streptococcus mutans*.

Groups	∆F (%)	∆Q (%mm^**2**^)	Lesion depth (µm)
Ss + Lc	-20.0 ± 2.6%^a^	-305.7 ± 54.3%^a^	137.5 ± 40.2^a^
Ss	-14.3 ± 3.1%^b^	-163.2 ± 85.6%^b^	84.1 ± 14.7^b^
Sm	-12.7 ± 2.9%^b^	-107.5 ± 54.4%^b^	55.0 ± 6.9^c^

*Different superscript letters indicate statistically significant differences among the different biofilm models (p<0.05).

**Figure 2:**
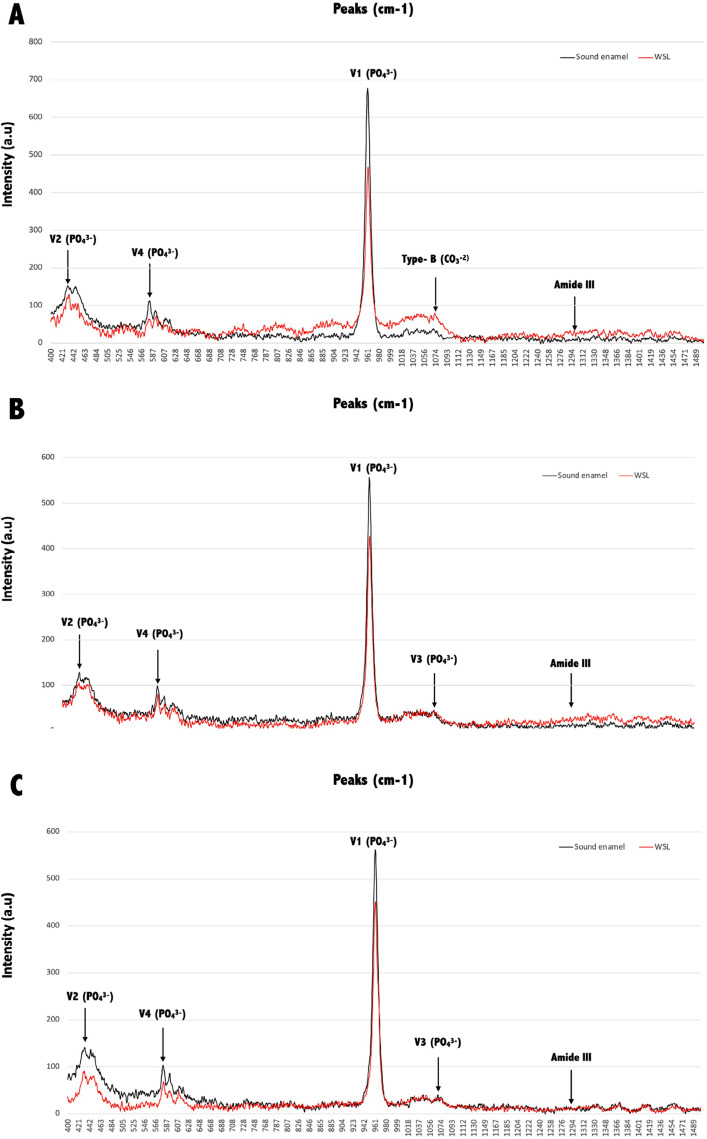
Spectra obtained by Raman spectroscopy from sound enamel (in black) and WSL (in red) created using the Ss+Lc (A), Ss (B), and Sm (C) biofilm models. Main peaks observed correspond to phosphate (PO4-3) vibrational bands v1 (phosphate symmetrical stretching mode) at 960 cm-1, v2 (phosphate symmetrical flexion vibration mode) at 431 cm-1 and 446 cm-1, v3 (asymmetric phosphate stretch) at 1,052 cm-1, and v4 (phosphate stretch mode) at 579 cm-1. Symmetrical stretching modes of type-B (1071cm-1) and type-A (1104 cm-1) carbonate (CO3-2), and amide III (1295 cm-1) are also observed.

Figure 2 shows the biochemical characterization results. Both Ss and Sm protocols showed the four phosphate (PO_4_^-3^) vibrational bands (*v*1=phosphate symmetrical stretching mode, *v*2=phosphate symmetrical flexion vibration mode, *v*3=asymmetric phosphate stretch, *v*4=phosphate stretch mode), while Ss+Lc showed only *v*1, *v*2, and *v*4. For all protocols, *v*1, *v*2, and *v*4 bands showed higher intensity for sound enamel than for the WSL, with the 960 cm^-1^ peak (*v*1) showing the highest intensity. When comparing the changes in *v*1 in WSL and sound enamel, this peak shows a decrease in WSL of 32%, 25%, and 20% for Ss+Lc, Ss, and Sm, respectively. Moreover, the symmetrical stretching modes of type-B (1069–1071cm^-1^) and type-A (1104 cm^-1^) carbonate (CO_3_^-2^) showed a higher intensity for WSLs than sound enamel only for the Ss+Lc protocol, demonstrating the substitution of phosphate by carbonate after the demineralization process. On the other hand, the A-type carbonate peak (1104 cm^-1^) was higher for sound enamel than for WSLs in the Ss and Sm groups. Regarding the organic content, the amide III (1295–1300 cm^-1^) peak showed higher intensity in WSLs than sound enamel for all protocols, with an evident increase in the lesions created using Ss+Lc.

**Figure 3:**
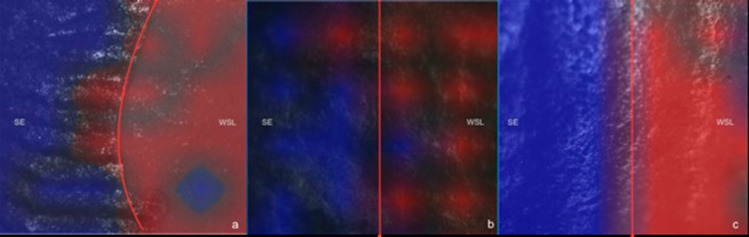
Raman mapping of the sample’s surface from protocols Ss+Lc (a), Ss (b), and Sm (c). Blue areas represent sound enamel (SE) and red areas indicate the white spot lesions (WSL).

Figure 3 shows representative images of the WSL surface obtained after the biofilm protocols. Raman mapping images show the two different zones, sound enamel (SE) and WSL, with a more or less pronounced transition between these two for the different protocols. All the red areas (WSL) showed a decrease in phosphate content and an increase in carbonate, in comparison to the blue side (SE) for all groups, as shown in the biochemical analysis. Blue spots can still be detected in some WSL areas, showing that each biofilm challenge provides a specific demineralization pattern that might not be homogenous.

## Discussion

The microbiological protocols tested in this study aimed to compare different bacterial strains and demineralization/remineralization cycles for proficiency in developing active WSL. All protocols created active WSL in enamel; however, there are differences in fluorescence, depth, and chemical composition when comparing the different models. Therefore, we reject the null hypothesis.

The selection of the bacteria for this study was based on their prominent role in caries disease. *S. mutans* and *S. sobrinus* are key cariogenic strains involved in caries lesion development in humans.^18^ These species stand out among plaque organisms for their extreme cariogenic properties, as they present strong sucrose-based adhesion due to water-insoluble glucan synthesis that promotes acid diffusion,^19^ prolific acid production from carbohydrate substrates, and exceptional acid tolerance.^20^ In addition, *S. mutans* is considered the acidogenic and aciduric strain responsible for synthesizing insoluble extracellular polymers using sucrose and promoting a plaque biofilm most conducive to lesion development.^15,18,21^

Simón-Soro, et al.^22^(2013) reported that *S. mutans* was not the dominant streptococci in enamel lesions, however, its proportion increased in plaque when compared to sound enamel. Even though some studies show contradictory results for the cariogenicity of *S. mutans* and *S. sobrinus,*^16,20^ our findings support the more pronounced acidogenic activity of *S. sobrinus* in comparison to *S. mutans*.^20^ This is confirmed by the significant demineralization resulting in increased lesion depth, changes in ΔF and ΔQ, and changes in the biochemical composition exhibited by the Ss+Lc and Ss groups.

In addition, *S. sobrinus* (in combination or alone) and *L. casei* were chosen to create biofilms combining the most acid-tolerant strains with the highest levels of acidogenicity. While S. *sobrinus* might not be as prevalent as *S. mutans*, the presence of these species correlates with greater severity and higher incidence of caries.^21,23^ Moreover, *L. casei* and *S. sobrinus* represent species that were previously shown to contribute to the progression of caries lesions.^24,25^ However, when comparing the composition of active bacterial strains in carious lesions, streptococci were detected as major species in enamel lesions and lactobacilli were clearly present in dentin lesions.^26,27^ Studies on the presence or absence of *Lactobacillus* species in plaque from WSL or cavitated enamel lesions are controversial. Some authors reported that *L. casei* could be detected covering WSL,^28^ although others showed that it appeared in high levels in deep caries in dentin, showing that they do present great acidogenic potential.^10,12^ Therefore, Lc was chosen to be used in combination with Ss to maximize the acidogenic potential of the bacteria in this protocol with the intention of either promoting deeper lesions or creating WSL more quickly than the other protocols, which was confirmed in our results. In general, the acidification of the environment can be one of the determinant factors in selecting the strains that promote demineralization for various tooth sites.^24^ Thus, the acidogenicity of the strains used in this study was crucial to obtain the WSL and agrees with previous reports using a combination of streptococci and lactobacilli for four days.^15^

Regarding the optical characteristics of the WSL created, there are some interesting aspects to be discussed. Lesions created by Ss and Sm presented similar ΔF and depth, which can be related to the same cariogenic activity presented by both strains.^16^ In contrast, the significant changes in ΔF and ΔQ in the Ss+Lc group reflect the more intense demineralization and higher scattering effect of the surface of WSL created with this protocol. Interestingly, ΔF values (20–25%) obtained for Ss+Lc in our study are similar to the reported for natural WSLs with QLF, which were shown to increase as the pitch angle increased.^29^ As already mentioned for the PLM images, WSL presented a slightly negative birefringence indicative of a more mineralized but not intact surface layer, which is a characteristic of natural WSL and a result of re-precipitation of calcium and phosphate ions released during the demineralization process.^30,31^ Interestingly, below this layer, we observed a positive birefringence for all groups and the natural lesion, which is characteristic of the body of the lesion. This region was where the most significant mineral loss occurred, resulting in increased pores and changes in the birefringence of the substrate in an aqueous medium (in this case, deionized water) as the pores are filled with water, which has been found in histology images of non-cavitated enamel lesions.^30,32^ Another important feature of artificial lesions is their shape.^30^ These lesions show a more regular appearance with their body lesion parallel to the enamel surface, which differs from the natural lesion format (convergent to the dentin-enamel junction) and is a result of the action of the slow speed attack during the demineralization and remineralization process. Even though this shape of artificial lesion has been reported previously, it was only possible after long incubation times such as 52 weeks using a chemical system.^30^ In our study, a long incubation time would make contamination likely and lead to surface erosion, which is one of the disadvantages of the microbial model and seen in our preliminary study.

Besides lesion depth and optical properties, this study also assessed the chemical composition and mapping of the WSL surface using Raman spectroscopy. The decrease in the phosphate (PO_4_^-3^) vibrational bands in WSL from all groups showed that all surfaces lost minerals, showing loss of crystallinity,^33^ while the remineralization was not able to recover the lost ions. The considerable change in the *v*1 peak in the Ss+Lc protocol agrees with the other results shown in this study, also indicating intense demineralization. In addition, the highest peak intensity for the symmetrical stretching mode of type-B carbonate (CO_3_^-2^) in the Ss+Lc group indicates substitution of phosphate with carbonate in the apatite lattice,^34^ known as B-type carbonate substitution. This modified carbonate hydroxyapatite is characterized by less stability, less hardness, and higher solubility to acid attacks than the other hydroxyapatites, making the surface more susceptible to acids.^33,34^ In addition, the type-A carbonate was also highest in the Ss+Lc group, demonstrating the higher representation of this band within the apatite lattice than the other groups, which reflects crystal imperfections associated with caries lesion development.^35^ While clear differences were observed for the inorganic content, further studies are needed to better understand the changes in the organic content observed during the early demineralization process, observed as the changes in the peak at 1295cm^-1^ (amide III) with higher intensity for Ss+Lc and Ss. In bone, this is related to tissue composition,^36^ and it may be related to changes in enamel due to caries lesions formation. Proteins such as amelogenins, the major enamel matrix protein related to amelogenesis and the structural organization of enamel, contain amide III and can be detected between the enamel crystallites on the sound enamel.^37,38^Thus, our data suggests that the demineralization may create gaps between the crystallites and expose the enamel proteins detected by the Raman readings.

Lastly, it is important to highlight that the biofilm models used in this study to create the WSL present some important advantages for mimicking caries progression. Creating an acquired pellicle before the incubation with the bacteria is a crucial aspect of this study. The affinities of the glucosyltransferases and extracellular polysaccharides to the tooth pellicle or the organisms themselves allow the biofilm to be firmly attached to the enamel surface. Moreover, the remineralization is another key step in the protocol since it aids to retain the lesion surface layer, as observed in the PLM. As already discussed, by performing the remineralization, surface erosion is avoided, mainly when protocols have to be performed for many days, which often leads to cavitation. Remineralizing solutions have been used in experimental chemical models, such as pH cycling, to simulate the natural demineralization/remineralization processes *in vivo*.^39,40^ These solutions contain various concentrations of fluoride to determine the degree of lesion progression.^17^ Previous studies indicate that high concentration of fluoride in the remineralization solution, such as 0.75 ppm, inhibited enamel lesion formation and decreased mineral loss by 30% to 40%.^8^ Thus, a limitation of performing remineralization, mainly when repeated multiple times (as in Ss and Sm groups), is that a very mineralized surface might be created on the enamel, protecting lesions from further progression and resulting in shallow lesions. Another limitation of the tested biofilm models is that even deeper WSL similar to the natural ones cannot be obtained since it would require extended cycles with the bacteria, possibly resulting in surface erosion and lesion cavitation, as already mentioned. We highlight that *in vitro* models do not reproduce entirely the complexities of the oral cavity. Therefore, we acknowledge that other bacteria strains and those related to caries lesion development, as well as multi-species models, should be further explored in future studies.

## Conclusion

In summary, despite the limitations of this *in vitro* study, all the microbial models were suitable for developing active WSL. The model using a combination of *S. sobrinus* and *L. casei* for four days produced active WSL with great depth, lower fluorescence, and significant changes in the inorganic content in a relatively short time. This protocol also preserved the surface layer and resulted in a subsurface lesion that mimics the characteristics of natural lesions. Overall, establishing an effective protocol for generating artificial lesions that mimic most aspects of *in vivo* lesion characteristics opens up many possibilities for laboratory studies to investigate novel and minimally invasive strategies to arrest lesion progression or treat aesthetic discolorations.
